# The impact of display saturation on visual search performance in congenital colour vision deficiency

**DOI:** 10.1371/journal.pone.0290782

**Published:** 2023-09-08

**Authors:** Amithavikram R. Hathibelagal

**Affiliations:** 1 Brien Holden Institute of Optometry and Vision Sciences, L V Prasad Eye Institute, Hyderabad, India; 2 Prof. Brien Holden Eye Research Centre, L V Prasad Eye Institute, Hyderabad, India; University of Tübingen, GERMANY

## Abstract

**Background:**

Colour-related search tasks are common in many professional fields. The study investigated whether increasing chromatic saturation can enhance the visual performance of individuals with colour vision deficiency (CVD) in colour-related search tasks.

**Methods:**

10 normal trichromats (5M, 5F; Mean (SD) age: 23.1 (3.3) years) and 15 individuals with CVD [8 deutans and 7 protans identified by HRR plates] (14M, 1F; aged 28.6 (8.7) years) participated in this study. Four naturalistic sceneries of everyday tasks/ birds, animals and flowers of 15 different colour combinations (1 pair of colours in each combination. e.g., ‘brown/black’ or ‘red/green’) were presented in ‘low’ saturation, ‘original’ (unaltered images) and ‘high’ saturation condition using the Psychopy program on a colour-calibrated monitor. On each trial, the subject was asked to identify a specific-coloured target.

**Results:**

Overall, the visual search performance index (expressed as product of accuracy and a reciprocal of reaction time (%correct*s^-1^) of the normal trichromats [Mean (SD):77.76% correct*s^-1^ (16.32)] was significantly higher than CVD [45.71% correct*s^-1^ (18.95)] in the “original” test images (p = 0.001), but in individuals with CVD, there was no significant difference between ‘original’ [45.71% correct*s^-1^ (18.95)] and ‘high’ saturation condition ([47.43% correct*s^-1^ (20.07)]; p > 0.05). However, colour-wise, increased saturation showed improvements (≥ 10%) in protans mainly for ‘red’ combinations with other colours such as white (i.e., ‘red/white’), purple, orange, grey, green, brown and black.

**Conclusion:**

The study suggests that increasing the saturation of certain colour combinations can potentially aid in the visual search performance of individuals with CVD. This knowledge will help in better counselling and management of the patients.

## Introduction

Colour-based visual search is a commonly encountered task in professions such as police [1], army, navy and other jobs in aviation [2]., Colour-dependent search can greatly improve efficiency [3, 4]. Individuals with inherited Colour Vision Deficiency (CVD) exhibit longer search times and lower accuracy in a colour identification [5] and colour-naming tasks [6]. Specific colour combinations such as yellow-green, and orange/red are more confusing to individuals with CVD [7]. However, lab-based visual search tasks that use specific-coloured target shapes (such as triangles or circles) do not reflect the suitability of an individual with CVD for a given profession. Thus, ‘occupation-based’ tests such as identifying coloured signals encountered in traffic [8] and colour-naming tasks in the visual display unit (used in railways) [9] are important. Colour-related visual search in a real-world activity such as searching for a red-coloured object on a grass lawn, [10] resembles the tasks encountered every day. However, presenting naturalistic images on a computer display allows for easier manipulation to study for a specific effect [11].

Increasing the chromatic saturation of the visual display may benefit individuals with CVD [e.g., reducing the task completion times [12]] because the threshold-level task for an individual with CVD is now potentially transformed into a suprathreshold task [5]. However, there is no strong evidence in the literature that either supports or refutes the hypothesis that altered chromatic saturation can affect visual search performance in individuals with CVD. Similar principles have been used in digital displays such as colour amplification and colour compensation in CRT and LCD monitors [13–15]. However, the visual performance of individuals with CVD in such manipulated or naturalistic images is limited and even more so in visual search paradigms. Only one previous study addressed this question, in which individuals with CVD identified specific features in 4 natural photographs (e.g., the ability to identify a red thread in a scenery of green shrubs) [16]. Chromaticity information regarding the target and distractor is missing. The colour combination assessed by Cole & Lian (2006) was restricted only to ‘red/green’ combination. However, Birch and Chisholm (2008) suggested several colour combinations that may be confusing to individuals with CVD [1]. Therefore, this study aims to assess whether changing the chromatic saturation alters visual search performance in naturalistic images across several colour combination pairs. If increased chromatic saturation improves the visual search performance, it can help them cope with the condition. In other words, individuals with CVD could potentially use increased saturation to better appreciate the colours and make adjustments accordingly in their digital and physical surroundings as much as possible. This new knowledge could potentially form the basis for further experiments on how the saturation of specific colours can be altered systematically to benefit individuals with colour vision deficiencies.

## Methods

### Participants

Twenty-five participants participated in the study. Out of which, 10 were normal trichromats (5M, 5F; Mean (SD) age:- 23.1 (3.3) years) and 15 individuals with colour vision deficiency [(14M, 1F; aged 28.6 (8.7) years)]. The screening plates of Hardy Rand Rittler (HRR) pseudoisochromatic plates (4th Edition 2002, Richmond Products, Albuquerque, NM, USA) distinguished normal trichromats from individuals with CVD, who were further tested using the Colour Assessment and Diagnosis (CAD) test (City Occupational Ltd, London) to identify the type and quantify the severity of colour vision defects. The test was performed according to the manufacturer’s instructions. The lighting for the HRR test was white light (standard office fluorescent lamp) with illumination of 300 lux. The individuals with CVD were subclassified as protans and deutans. All participants had a monocular visual acuity of at least 20/20 (logMAR– 0.0) with refractive correction in place. The participants were sourced primarily from patients who reported to the L V Prasad Eye Institute (LVPEI), Hyderabad campus. Individuals with CVD in staff and student cohorts were also recruited for the study. The study was conducted at the L V Prasad Eye Institute, Hyderabad. The participants were informed of the test duration and nature of the test. If the participants were interested, written informed consent was obtained prior to testing. This study adhered to the tenets of the Declaration of Helsinki. The study protocol was approved by the Institutional Review Board of the L V Prasad Eye Institute, Hyderabad (Reference no: LEC-BHR-P-09020-509).

### Stimulus

Specific natural images (such as scenery, flowers, fruits, birds, animals, and everyday tasks) were chosen from Flickr (https://www.flickr.com/explore) and 500px (https://500px.com/) websites that represented 15 colour combinations: brown/black, red/purple, red/green, red/white, red/brown, red/orange, red/grey, red/black, green/yellow, green/blue, green/white, green/brown, green/grey, orange/brown, and orange/yellow. The colour combinations were chosen based on the common colours confused by individuals with CVD [1]. The images were considered representative of any of the predetermined specific colour combinations, based on the proximity of specific colours in the visual scene.

**Fig 1:**
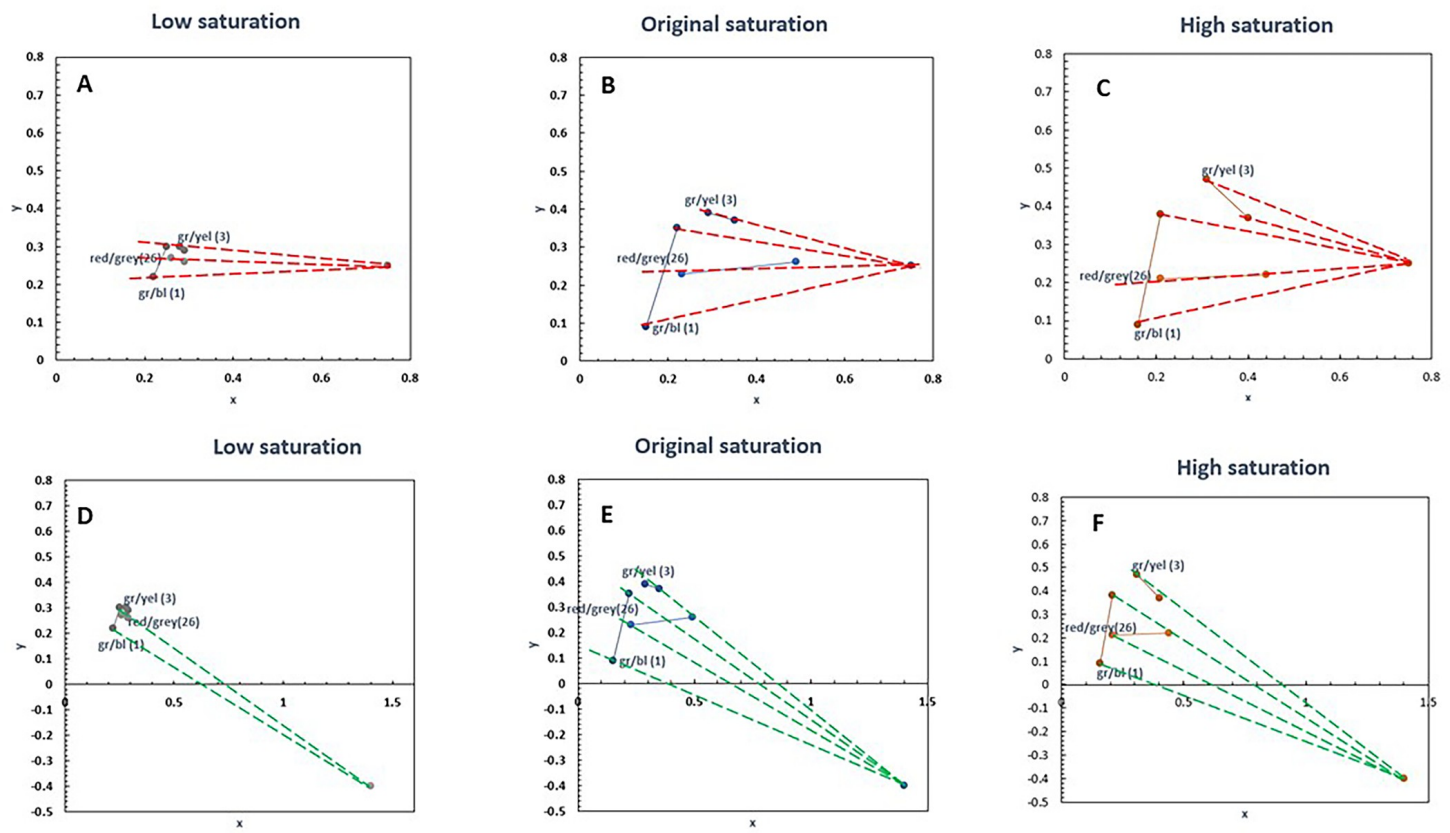
CIE Plots showing specifications for one set of 3 colour combinations in all three saturation levels (‘low (left panels), ‘original’ (middle panels) and ‘high’ saturation (right panels) condition in 1931 CIE space. The top panels include the colour confusion lines for a protanope derived from drawing a line from a given chromaticity coordinate to the copunctal point (0.75,0.25). Similarly, the bottom panels refer to the colour confusion lines for a deuteranope [copunctal point–(1.4, -0.4)] [27]. The number in the parenthesis refer to the image number. The labels corresponding to the abbreviations are as follows: gr/bl–green/blue; gr/yel–green/yellow;; red/grey.

The chromaticities of the combination colours for all the images in the ‘original’ (unaltered condition) are provided in the CIE 1931 colour space (S1 Data). The tristimulus values were using an XRite Colorimeter (i1Display Pro model, XRite, Incorporated, Germany), which measured the chromaticity on the LCD monitor. The third-party software for use of colorimeter was provided by https://www.argyllcms.com/. The tristimulus values were then converted to CIE xyz values using well-known conventions [17] The CIE 1931 x and y values for each colour pair in all the images are provided in S1 Data (Fig 1 for one set of 3 colour combinations at all three levels, namely low, original, and high saturation). The images were presented using a Psychopy program (Version 2.6) [18], which was run on a laptop connected to a 24” colour-calibrated LED-backlit LCD monitor (EIZO, Model Color Edge CS2420; EIZO Corporation, Japan). The display is embedded with a 10-bit graphic card whose resolution was 1920 X 1200 pixels with a frame rate of 60 Hz. The ‘white’ setting of the monitor is the MacAdam white (0.32, 0.33) which is very close to D65 (0.31,0.33). The gamut of colours that can be represented in the monitor is shown in S1 File. The display monitor was separated from the laptop using a black curtain to avoid distractions for the participants. The size of the image was 15 cm × 15 cm subtending 8.6° at 1m distance. The saturation of the images was manipulated using the GNU Image Manipulation Program software (GIMP Version 2.8.16; https://www.gimp.org/.) The saturation level was set at -75% for ‘low’ saturation and +75% for the ‘high’ saturation category (See example figure in S2 File). Three examples of images with different saturation levels of in CIE colour space are shown Fig 1. The chromatic displacement (distance between a given pair) increases with an increase in saturation. The exact setting change made in the GIMP software used to change the saturation is provided in S2 File. There was a total of 60 images (4 images ×15 colour combinations) presented in each saturation condition (‘low’, ‘original’ [unaltered image set] and ‘high’). Therefore, the participants responded to a 180 images (60 images × three levels of saturation). Each colour combination set consisted of four different images, and each image was distinct from the others in the given set and from the entire set of images. The image count is displayed in the right corner above the instructions for each image. The order of the images in each block under the ‘saturation’ condition was randomised. However, the sequence of presentation blocks was as follows: ‘low’ saturation category, followed by ‘original’ images, and then ‘high’ saturation images. This sequence was performed to avoid memorising specific distinct features in an image, which is likely to be more vivid in the ‘high’ saturation category. The participants were naïve to the purpose of the experiment. They were instructed only to perform an accurate visual search task as fast as possible based on the instructions provided in the respective images. The participants were not asked to prioritise either accuracy or speed in the given instructions.

**Fig 2:**
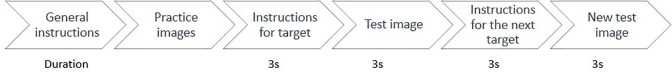
Timeline sequence of the events during the experiment.

The instructions for identifying the specific ‘target’ in each image were provided as a ‘text’ for 3 seconds before the image was presented. The duration of each image was 3 seconds. (Fig 2 for the timeline of presentations). The text instructions continued to remain until the image disappeared, which happened either after a mouse click on the image or 3s after the image onset.

### Procedure

Participants performed the visual search task binocularly in a dim-lit room. None of the participants wore tinted spectacles or contact lenses. Participants were seated 1m distance from the display monitor. Participants were instructed to correctly identify the targets as soon as possible. There were eight practice presentations that served as examples during which the participants became accustomed to the task. Only the ‘original’ saturation images were used in the practice session. The practice images were different from those used in the primary experiment. Irrespective of whether the participant responded within three seconds, the test proceeded further. No feedback was provided to the participants regarding the correctness of their responses. The position of the mouse pointer was recentered to the centre of the screen ([0, 0]; Cartesian coordinates) after each mouse click. There was a 30-second break after every 30 trials to prevent fatigue. The total duration of the test was approximately 18–20 minutes. The display monitor was screenrecorded using the Camtasia recorder (Camtasia, Camtasia Studio, Version 7.1.1, TechSmith Corporation, USA), which was later analysed offline to identify the correct responses in each of the presentations.

### Data analysis

Visual performance was characterised by the reaction time and accuracy. Accuracy was defined as the percentage of correct responses in the total presentations. i.e., participants making correct mouse clicks within ± 0.5 cm from the edge of the region of interest. The time taken to click on the target in the image since the onset of the presentation was defined as reaction time, which was tabulated in the Psychopy output file. The reaction time was averaged only for correct responses. The visual search performance index was computed as the product of accuracy and the reciprocal of the reaction time for the correct responses (in seconds). The data were normally distributed according to the Shapiro-Wilk test. One-way ANOVA was performed on the outcome variables (accuracy, reaction time, and performance index) in normal trichromats to study the effects of different levels of saturation (low, original, and high). Similarly, one-way ANOVA was performed in each group of CVD (protans and deutans)) to study the effect of the three levels of saturation on the accuracy, reaction time, and visual search performance index. The interactions between the two types of defects and the levels of saturation were also studied. For the post hoc test, the significance for p-values involving three comparisons (low, original, and high saturations) was set to less than 0.016 (0.05/3), after applying either Bonferroni correction, or a Tukey HSD test. A 10% decrease or increase in performance relative to the ‘original’ condition was considered worsening and improvement, respectively. This value was chosen based on the relative variation in the performance of normal trichromats with different levels of saturation.

## Results

The mean R-G CAD threshold across all individuals with CVD was 25.1 (9.18) CAD units. The higher the CAD test results, the worse is the colour vision. The CAD test results were available for 14 of the 15 individuals. There was no significant difference in the severity of scores between deutan (26.30 (3.98) CAD units) and protans (23.98 (12.82) CAD units; t-test, p = 0.61). The coefficient of variation (COV) is higher in protans (0.53) than in deutans (0.15). The profiles of the individuals with CVD and their CAD test results are presented in Table 1. The severity of CVD status was classified based on the norms proposed by Barbur et al. (2017) [5].

**Table 1:** Profile of individuals with CVD along with type and severity of CVD based on CAD threshold and grading system[5].

Subject	Age	Gender	CAD thresholds	CAD Severity	CVD type
S1	23	M	26.6	Severe	Deutan
S2	30	M	31.5	Severe	Deutan
S3	20	M	NA	---	Deutan
S4	14	M	25.7	Severe	Deutan
S5	40	M	31.2	Severe	Deutan
S6	38	M	21.1	Severe	Deutan
S7	35	M	25.8	Severe	Deutan
S8	36	M	22.3	Severe	Deutan
S9	25	M	15.5	Severe	Protan
S10	32	M	47.9	Severe	Protan
S11	20	M	8.1	Poor	Protan
S12	31	M	28.0	Severe	Protan
S13	30	M	21.2	Severe	Protan
S14	40	M	17.8	Severe	Protan
S15	15	F	29.3	Severe	Protan

Among the different levels of saturation in normal trichromats, the accuracy and performance index showed significance (p < 0.05), whereas reaction time showed no significant change (p = 0.07). Further, on post hoc testing (Tukey HSD), it was revealed that accuracy in the ‘original’ saturation (Mean ± SD:98.33 ± 2.04%) category was significantly higher than the ‘low’ saturation category (93.67± 6.04%; p = 0.007). Similarly, on post hoc testing (Tukey HSD), the performance index in ‘original’ saturation (82.96 ± 6.17% correct*s^-1^) category and ‘high saturation (83.28 ± 7.46% correct*s^-1^; p < 0.05) was significantly higher than the ‘low’ saturation category (71.76 ± 9.88% correct*s^-1^; p < 0.05) respectively.

The overall visual search performance index in normal trichromats (mean (SD): 77.8% correct*s^-1^ (16.3)) was significantly higher than that in CVD (45.7% correct*s^-1^ (19)) in the “original” test images (p = 0.001). A 2-factor repeated measures ANOVA (type of defect × saturation) revealed that the main effect of saturation on the visual search performance index was statistically significant (F (2, 647) = 23.23, p <0.0001, effect size [η_p_^2^ = 0.07]). Further post-hoc analysis using Bonferroni correction, with regard to saturation, revealed that the visual search performance index in both the ‘original’ and ‘high’ saturation conditions was significantly better than the ‘low’ saturation performance condition in individuals with CVD. However, there was no significant difference between the ‘original’ (45.17% correct*s^-1^(9.44)) and ‘high’ saturation condition (46.9% correct*s^-1^(10.52); p = 0.71) in individuals with CVD.

In addition, the main effect of the type of colour vision defect (protan/deutan) on the visual search performance index was significant (F (1, 647) = 26.8, p <0.0001, η_p_^2^ = 0.040), and protans performed better than deutans. There was also a significant interaction between saturation and defect type (F (2, 647) = 4.7; p = 0.009, η_p_^2^ = 0.02). Similar patterns were observed for other visual search parameters, such as accuracy and reaction time, except for the absence of a significant interaction between saturation and type of defect. Overall, compared to deutans, all outcome variables of protans were significantly better (shorter reaction times, and greater accuracy and visual search performance index) (Tables 2 and 3).

**Table 2:** Shows ANOVA 2- way results summarizing the main effect of type of colour vision defect (protan/deutan) on each of the outcome variables (accuracy, reaction time and performance index) for data grouped across the three levels of saturation.

Outcome variables	Type of defect		Mean difference (s)	Lower limits	Upper limits	F value	p-value
REACTION TIME	Deutan Mean (s)	Protan Mean(s)					
Deutan–protan	1.81 (0.16)	1.69 (0.17)	0.12	0.05	0.19	12.82	<0.001**
ACCURACY	Deutan Mean (%)	Protan Mean (%)	Mean difference (%)	Lower limits	Upper limits	F value	p-value
Protan—Deutan	66.14 (12.36)	73.57 (13.33)	7.43	2.78	12.09	10.08	0.002*
PERFORMANCE INDEX	Deutan Mean (% correct*s^-1^)	Protan Mean (% correct*s^-1^)	Mean difference (% correct*s^-1^)	Lower limits	Upper limits	F value	p-value
Protan—Deutan	38.64 (9.14)	46.55(10.47)	7.90	4.31	11.50	19.09	<0.001**

**Table 3:** Shows ANOVA 2- way results summarizing the main effect of levels of saturation (low, original and high) on each of the outcome variables (accuracy, reaction time and performance index) in individuals with CVD.

Outcome variables		Saturation levels			Mean difference	Lower limits	Upper limits	F value	p-value
		Low	Original	High					
REACTION TIME		Main effect						3.67	0.03*
Saturation	low-original	1.81 (0.17)	1.70 (0.18)	----	0.11	0.01	0.21		0.03*
	low-high	1.81 (0.17)	----	1.73 (0.18)	0.09	-0.02	0.19		0.11
	high-original	----	1.70 (0.18)	1.73 (0.18)	0.023	-0.08	0.12		0.85
ACCURACY		Main effect						15.96	<0.001**
Saturation	original-low	60.64 (11.55)	73.09 (11.62)	----	12.46	5.62	19.30		<0.001**
	high-low	60.64 (11.55)	----	75.83 (11.85)	15.2	8.35	22.04		<0.001**
	high-original	----	73.09 (11.62)	75.83 (11.85)	2.74	-4.10	9.58		0.61
PERFORMANCE INDEX		Main effect						14.69	<0.001**
Saturation	original-low	35.73 (8.20)	45.16 (9.44)	----	9.42	4.14	14.71		< 0.001**
	high-low	35.73 (8.20)	----	46.9 (10.52)	11.16	5.88	16.45		<0.001**
	high-original	----	45.16 (9.44)	46.9 (10.52)	1.74	-3.54	7.03		0.71

* indicate significance at the level of 0.05 and

** indicates significance at the level of 0.001.

### Colour-wise visual performance

**Fig 3:**
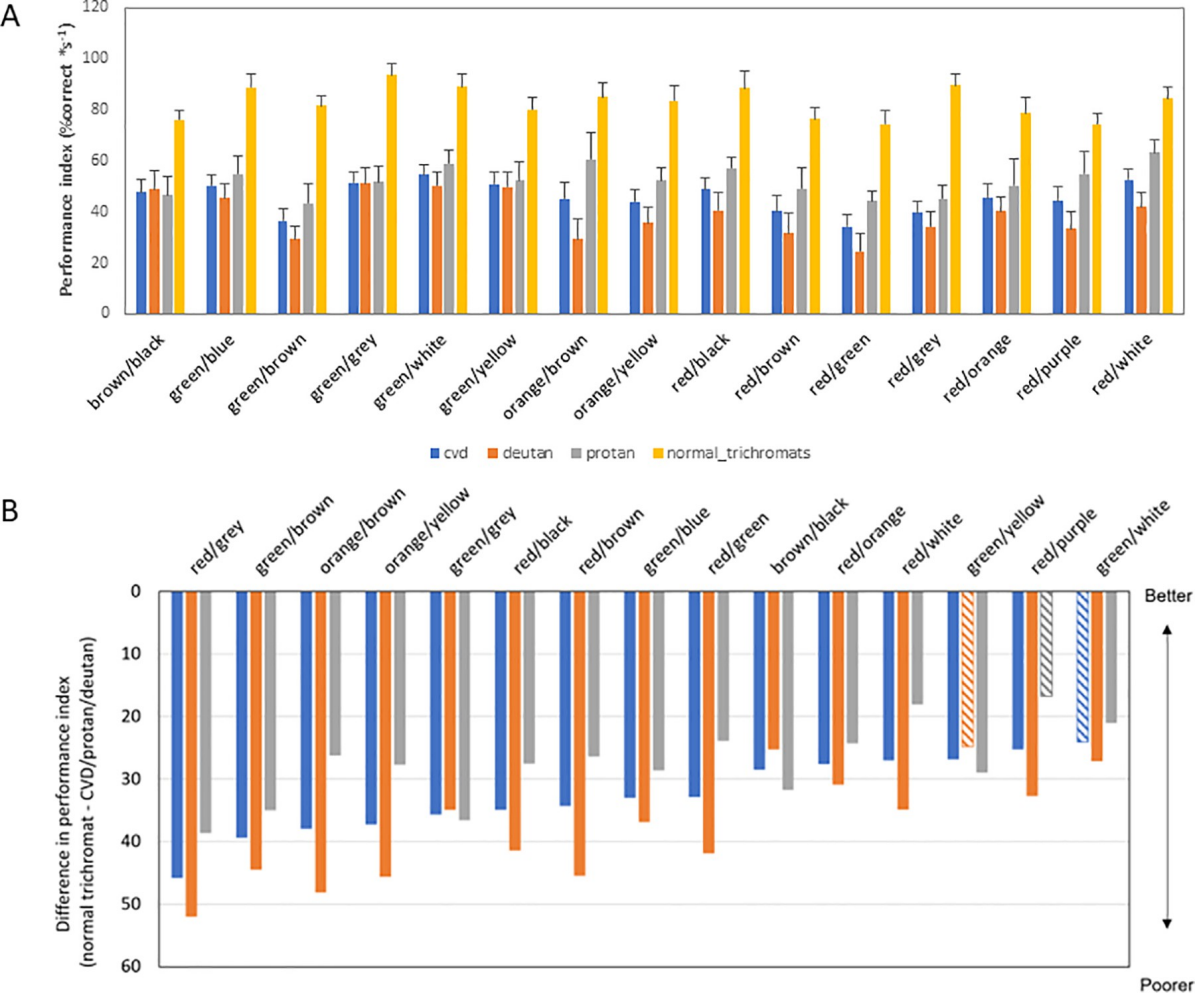
Average visual search performance index along the different colour combinations in the ‘original’ category for all the two main groups (normal trichromats and individuals with CVD). The indices are also provided for subgroups of CVD namely protan and deutan. The square symbol indicates the average visual search performance index of normal trichromats. The error bars indicate the standard error of the mean. The upright triangles indicate the visual search performance index of the individuals with CVD. The diamonds and inverted triangles indicate the visual search performance index of the protans and deutans respectively. Protans outperform deutans for the majority of colors. Panel B shows the differences in performance index with regards to various colour combinations in individuals with CVD (as a group as well as subgroup) relative to normal trichromats. The higher the difference, the worse is the performance for that combination for that group. ‘Red/grey’ combinations appear most difficult to the individuals with CVD while ‘green/white’ task appear the easiest to cope with.

The minimum and the maximum difference in the visual search performance index of the individuals with CVD compared to the normal trichromats was noted in the ‘green/white’ and ‘red/grey’ combination respectively (Fig 3A and 3B). In protans and deutans, similarly, the maximum difference in visual search performance index relative to normal trichromats was noted for the ‘red/grey’ combination. The minimum differences compared to normal trichromats for protans and deutans were noticed in ‘red/purple’ and ‘green/yellow’ respectively. The maximum visual search performance index in the CVD cohort was noted for the ‘green/white’ and the minimum was the ‘green/brown’ combination among all the combinations. The maximum visual search performance index in the protans was noted for the ‘red/white’ and the minimum was the ‘green/brown’ combination among all the colour combinations. The maximum visual search performance index in the deutans was noted for the ‘green/white’ and the minimum was the ‘red/green’ combination amongst all the colour combinations.

**Fig 4:**
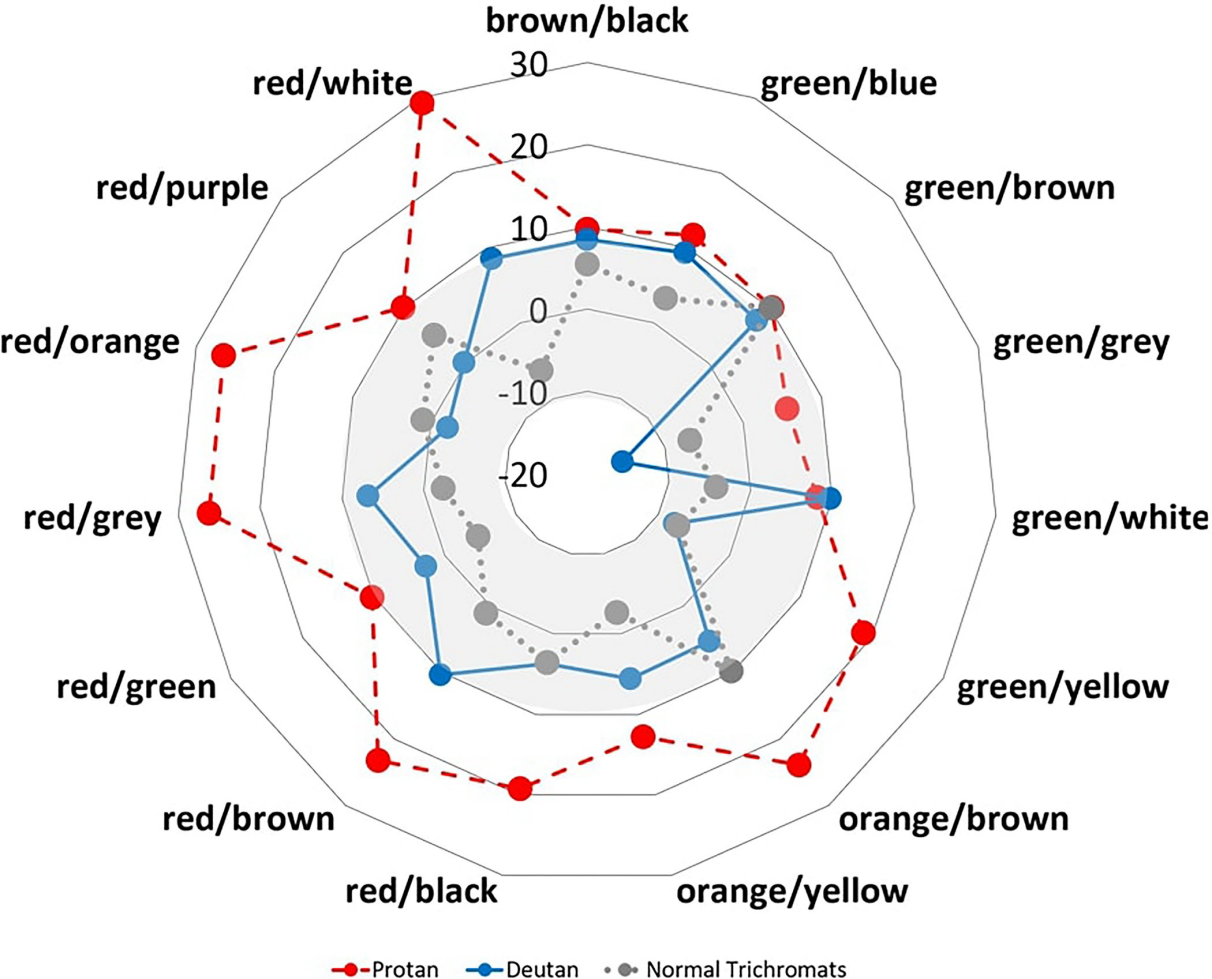
Polar plot showing differences in visual search performance index (‘high’ saturation–‘original’ condition) for all the colour combinations for both protans (red-dashed lines) and deutans (blue solid lines). The negative numbers in the polar plot refer to worsening effect and the positive numbers indicate a positive or improvement. On increasing the saturation of the display, improved visual search performance index for most combinations was noted for protans than deutans. The grey zone (mid central rings) is considered a region of “no change”, as the change in visual search performance index is ≤ 10%.

The normal trichromat performance in the ‘high’ saturation category was not significantly enhanced compared to the ‘original’ category. In protans, there were numerous colour combinations (red/white, red/purple, red/orange, red/grey, red/green, red/brown, red/black, orange/yellow, orange/brown, and green/yellow) which improved upon increasing chromatic saturation (Fig 4). However, in deutans, only the red/brown combination improved with an increase in chromatic saturation. There was no significant correlation between visual search parameters (accuracy, performance, and reaction time) and the CAD severity score in any of the conditions (r ≤ 0.35; p >0.05).

## Discussion

There are four key findings of this study. First, the visual search performance index in individuals with CVD was significantly poorer compared to normal trichromats (Table 1). Second, protans performed better than deutans in ‘original’/unaltered test conditions (Fig 3A and 3B). Third, the effect of increasing saturation in the improvement of the visual search performance index is dependent on the type of colour vision defect and the type of specific colour combinations. Lastly, there was a lack of association between outcome variables and the severity of the colour defect.

The poor visual search performance index of individuals with CVD relative to normal trichromats is consistent with several other previous studies [8, 9, 16, 19]. The results agree with the study by Cole and Lian (2006) who assessed visual search in individuals with CVD using natural scene photographs. Only accuracy was reported in the study by Cole and Lian (2006) [16]. Therefore, other parameters, such as reaction time and the visual search performance index cannot be compared.

Overall, the visual search performance of individuals with protan deficiency was better than that of individuals with deutan deficiency. The visual search task in this study was not a threshold-level task. It is possible that starting ‘chromatic saturation’ used in the ‘original’ image condition may itself be representative of a suprathreshold task, where protans perform better than deutans [20]. Besides, luminance is altered when the saturation is varied, therefore the altered parameter can be broadly referred to as “colourfulness” instead of only chromatic saturation. These luminance cues could have helped the protan participants. Previously in a couple of studies by Cole et al. they have found that protans fare better than deutans in natural scenes [16] and also in tasks involving visual search in a laboratory-based task [19]. However, in another visual search study by Cole (1988) [21] on individuals with CVD on colour-coded video displays, they noticed that protans were slightly worse than deutans in several tasks and were also slower to respond. The discrepancies could be attributed to the variability in the tasks encountered in each study and the severity of the defect variation across the studies.

Chromatic displacement is a concept used in the CAD test as well, where the target and background are initially separated by a small chromatic displacement in the CIE colour space which is either increased or decreased based on the performance of the individual. Increasing the saturation should make the task easier. However, there is a caveat: this improvement is noticed only when the chosen colours are in different confusion lines. This may also explain why the effect of increasing chromatic saturation did not improve the visual search performance index for some colour combinations. There are few specific colour combinations where the visual search performance index worsens (e.g., green/grey in deutan subjects; Fig 4). Overall, a minimum performance in the cohort of CVD was noted in the ‘green-brown’ combination. Protans also performed poorly in the ‘green-brown’ combination. This is consistent with a previous report [7], as it involves the combination of both colours which may lie on colour confusion lines. The maximum performance was noted in the white combinations for both subgroups, which has larger chromatic displacement between target colour and white (0.33, 0.33). The combination of colours might make the visual search task easier, provided they do not lie on the confusion lines. Here is an example that illustrates this point. Please refer to Fig 1B and 1C for colour combination gr/yel(3). As the saturation increases, gr/yel (3) is no longer on the same protan confusion line (panel C) as earlier (panel B). However, in panels E and F, the colour combinations continue to be present near the deutan confusion line. Therefore, an improvement in performance (Fig 4, polar plot) is noted for protan and not for deutan for the gr/yel combination. Also, depending on the initial saturation of the image and the initial chromatic displacements, some pairs may be more difficult compared to others.

There was a poor correlation between the CAD test scores and outcome variables in this study. This is partly in agreement with a previous study which showed that beyond 10 CAD units, there is no clear relationship between the severity scores and time taken to complete the colour identification tasks [12]. CAD test does not distinguish anomalous trichromacy from dichromacy. The lack of correlation could be attributed to the fact that the images may be suprathreshold, which is unlike the testing performed in the CAD test. Therefore, threshold- level results may not represent suprathreshold performance, and how occupational requirements for colour should probably not be based on threshold-level performance. Besides, the lack of correlation could be attributed to other factors such as eye movements and other attention and search strategies playing a role in visual search [22].

As CVD is not treatable [23] therefore there is a larger emphasis on developing useful coping strategies for this group and also on whether deficiency-specific managing advice can be generated [24]. The results from this study indicate that for specific colour combinations, the visual search performance index of individuals with CVD can be significantly improved by increasing chromatic saturation. For example, there are specific colour combinations used (red/white) in the aviation industry that indicate the specific state of function [5]. The results of this study suggest that, in principle if these coloured objects in their ‘original’ state are replaced with identical objects albeit with highly saturated colours, it may potentially help in the perceptibility of the targets for individuals with CVD. However, the results must be interpreted with caution, as the images used in the study did not have equal difficulty levels, and luminance contrast changes may not be uniform across levels of saturation. A similar principle has worked in the microbiology field, where red features in an image can be replaced by magenta colour and visibility improves dramatically in individuals with CVD [25]. With a commercially available portable spectrometer (Photonfy; https://ledmotive.com/photonfy/), it would be relatively easier to estimate the chromaticity of the targets in the realworld and ensure that modified coloured targets are of specified recommendations. However, the results of this study cannot be generalised to objects in the real world. Nevertheless, it provides a framework regarding which colour combinations can be potentially modified to enhance the visual search performance index in individuals with CVD.

The present study has some limitations. The selection of individuals included almost an equal percentage of protan and deutan deficiencies which is inconsistent with the prevalence of CVD [26]. However, this could be attributed to the differences between population-based screening studies and lab-based small sample cross-sectional studies. With regard to methods, usage of desaturated colours may delay the reaction time even in individuals with normal trichromacy as the colours may be slightly misrepresented (e.g., desaturated red may look pink) and therefore might result in a slightly longer reaction time or poorer accuracy. However, overall, normal trichromats consistently performed better than individuals with CVD did. The target size and difficulty of the task were not uniform across all the combinations. There were some tasks where there were more than one/two distractor colours in the scenery along with the target colour and that would be considered a relatively more difficult task [19]. Besides, when the saturation is enhanced, there is also a change in photopic and scotopic luminance, which may affect the visibility of the targets differentially. The asymmetry in colour differences in certain colour combinations (e.g., red/white) unlike others (for example; red/brown) may also affect the perceived difficulty of the task [5]. The images in which there are larger colour differences are likely to result in a more accurate performance, even in individuals with moderate CVD. In a few images of visual search, ‘top-down’ influence in reaction time and accuracy cannot be ruled out. Also, the colour identification task in the visual search paradigm is largely dependent on colour naming by individuals with CVD, which can be flawed sometimes [6]. However, real-world situations provide no such concessions, therefore, it is important to assess the visual search performance index even in the face of apparent confounding factors such as colour naming. Besides, owing to the limited sample size of the study, one must be careful before generalizing conclusions to a larger population.

## Conclusions

The study concluded that increased chromatic saturation produces a differential effect across various chromatic combinations, especially protans benefitting from higher saturations in most combinations containing red. Therefore, this study is the first step towards potentially modifying colour-specific environmental settings in an occupational environment based on increased chromatic saturation to aid individuals with CVD.

## Supporting information

S1 Data Sheet containing CIE xy data regarding the colour combinations. The combinations highlighted in yellow are demonstrated in Fig 1.
(XLSX)

S1 File Gamut of colours that can be shown in the testing monitor. (PDF)

S2 File Use of supplemental figure to show GIMP software was utilized. (PDF)
