# Supplementary material for: The impact of display saturation on visual search performance in congenital colour vision deficiency
Source: PLoS One. 2023 Sep 8;18(9):e0290782. doi: 10.1371/journal.pone.0290782 (PMC10490843; doi:10.1371/journal.pone.0290782)
Supplement: S1 File — (PDF) [file pone.0290782.s002.pdf]

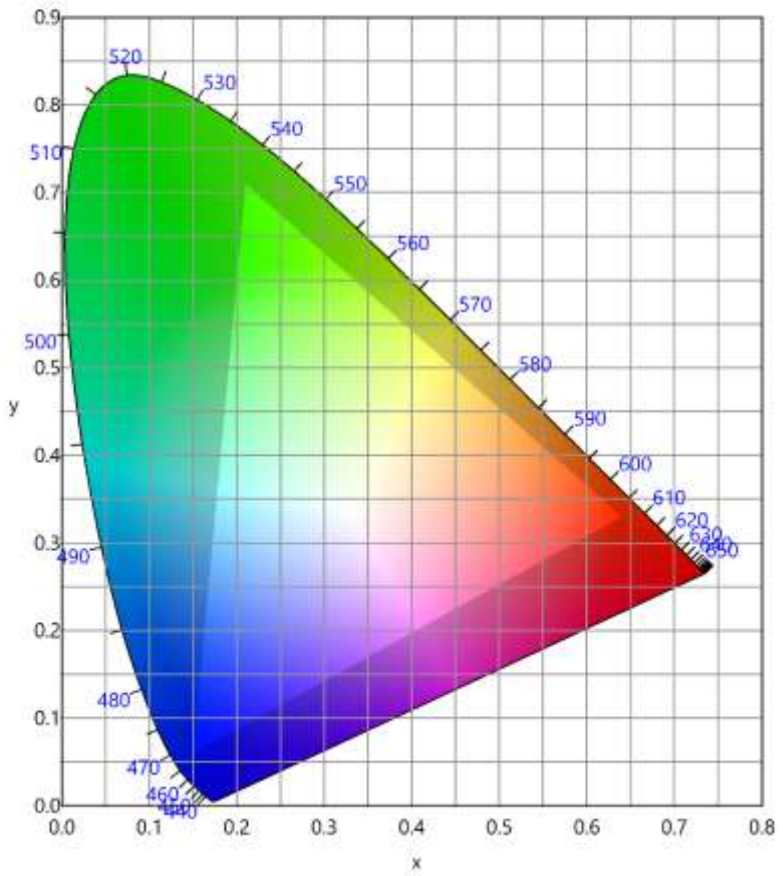

Figure S2 showing the gamut of colours in the CIE 1931 space that can be produced on the testing monitor. The triangle represents the gamut of colours. The gamut was derived based on the company specifications.
