# Supplementary material for: The impact of display saturation on visual search performance in congenital colour vision deficiency
Source: PLoS One. 2023 Sep 8;18(9):e0290782. doi: 10.1371/journal.pone.0290782 (PMC10490843; doi:10.1371/journal.pone.0290782)
Supplement: S2 File — (PDF) [file pone.0290782.s003.pdf]

‘Low’ Saturation

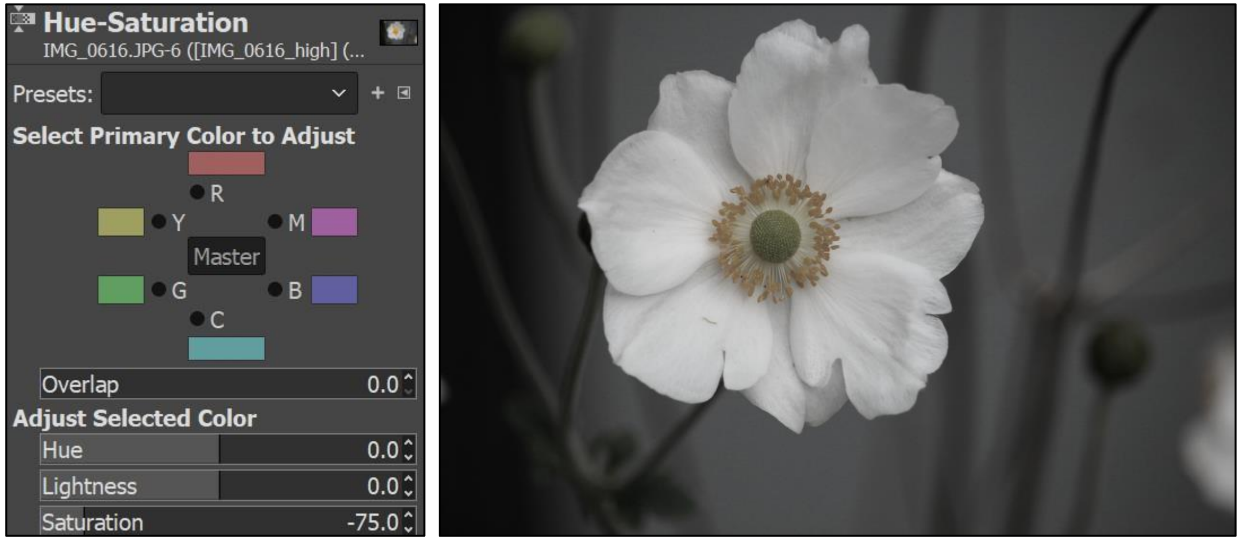

‘High’ Saturation

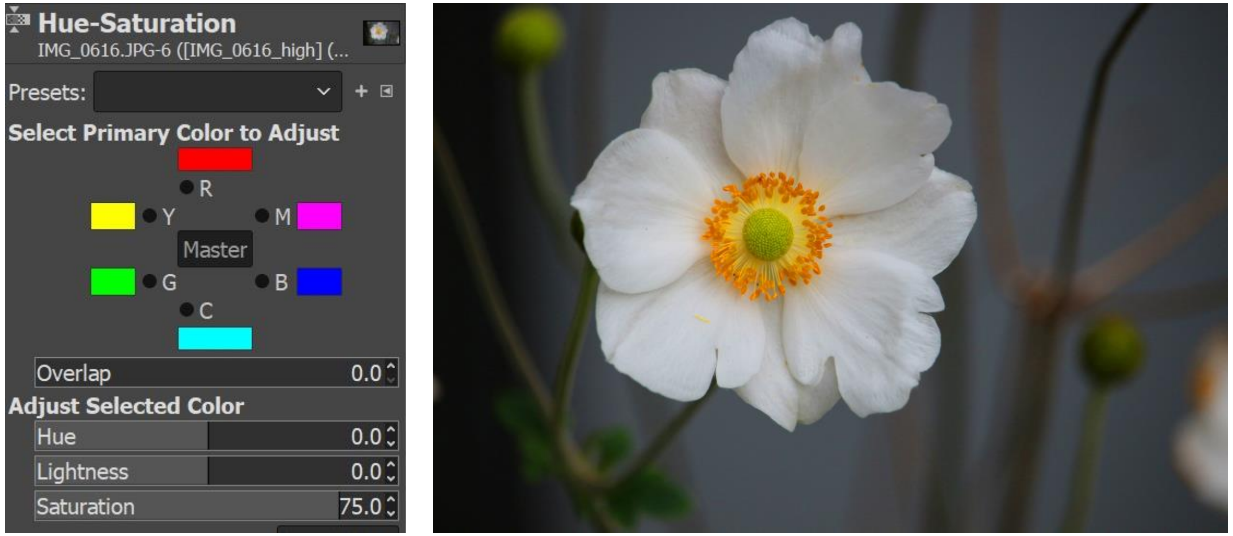

‘Original’ Saturation

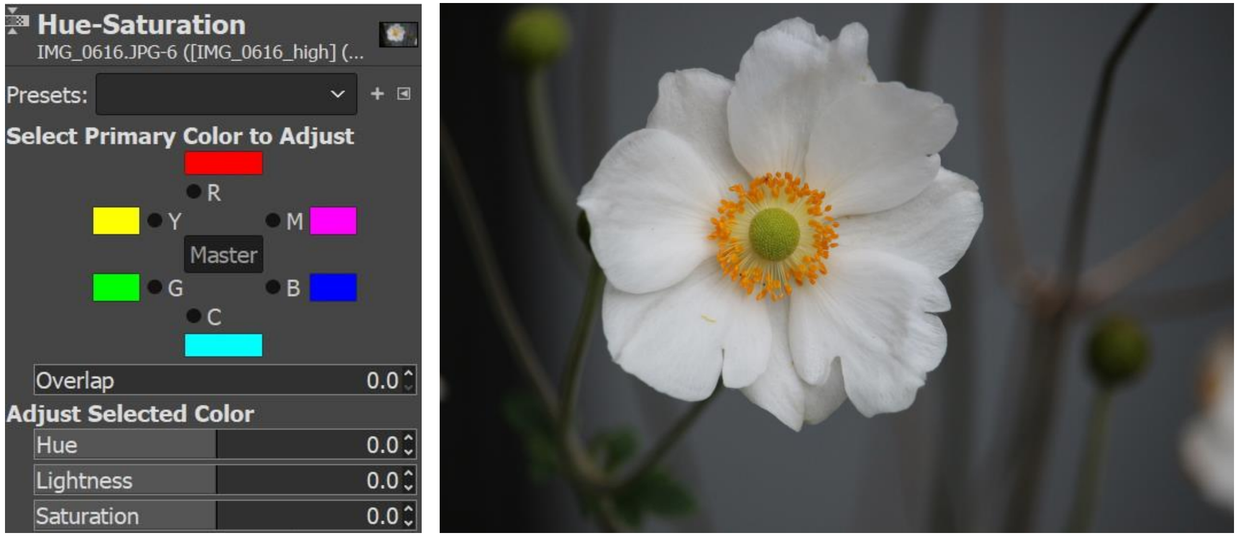

Figure S3 showing the settings used in GIMP software to generate ‘low’ and ‘high’ saturation categories as shown above. The middle panel shows the saturation value = 0, which is the original image or unaltered image.
